# PRMT5 genetic interactions with DNA double strand break repair genes

**DOI:** 10.1371/journal.pone.0331499

**Published:** 2025-10-09

**Authors:** Hunter J. Bliss, Juliana Tron, Wesley Bush, Renee A. Bouley, Ruben C. Petreaca

**Affiliations:** 1 Biology Program, The Ohio State University, Marion, Ohio, United States of America; 2 The James Comprehensive Cancer Center, The Ohio State University, Columbus, Ohio, United States of America; 3 Department of Chemistry and Biochemistry, The Ohio State University, Marion, Ohio, United States of America; 4 Department of Molecular Genetics, The Ohio State University, Marion, Ohio, United States of America; Tulane University Health Sciences Center, UNITED STATES OF AMERICA

## Abstract

Protein arginine methyltransferase 5 (PRMT5) has pleiotropic functions in human cells but also participates in orchestrating DNA double strand break (DSB) repair. It methylates the TIP60 histone acetyltransferase complex to facilitate recruitment to the DSB and chromatin remodeling. PRMT5 mutations affect DSB repair by homologous recombination and increase chromosomal instability. In this report we characterized genetic interactions between PRMT5 mutations and mutations in other components of DSB repair pathway. We used data deposited on the Catalogue of Somatic Mutations in Cancers (COSMIC). We found that PRMT5 makes negative genetic interactions with TIP60 and member of the 9-1-1 complex (RAD9, RAD1, HUS1) which is required for checkpoint activation. A comprehensive analysis of all cancer data deposited on COSMIC reveals very few samples with mutations in both PRMT5 and TIP60 or components of the 9-1-1 complex in samples where mutations in other DNA damage repair genes occur (e.g., MRN, checkpoint genes, etc). This suggests that when more factors of the DNA damage repair machinery are destabilized, the functions of TIP60 and 9-1-1 appear to become essential. Protein 3-D structure analysis shows that mutations affect protein-protein interactions that may destabilize 9-1-1 or TIP60 complex formation. These data highlight interesting interactions between the various genetic pathways governing DSB repair. It also reveals potential therapeutic targets. For example, inhibition of the 9-1-1 complex in a PRMT5 mutant may selectively kill the cell. Given that PRMT5 small molecule inhibitors are being developed or already deployed, these findings should inform potential applications of these drugs.

## Introduction

Repair of DNA *D*ouble *S*trand *B*reaks (DSBs) is a complex process that requires the interplay of several cellular activities such as activation of the DNA damage checkpoint, chromatin remodeling, recruitment of the DNA damage repair machinery, and re-deposition of histones to preserve epigenetic markers [1–3]. In eukaryotes, DSBs are repaired by two major but related mechanisms: *N*on-*H*omologous *E*nd *J*oining (NHEJ) and *H*omologous *R*ecombination (HR) [4,5]. The choice between the two pathways is primarily determined by the stage of the cell cycle [6–8].

Accurate chromatin remodeling at DSBs is essential to promoting efficient repair [9]. Mutation in chromatin remodeling factors cause genomic instability which leads to cancer [10,11]. Histone acetylation is key to relaxing chromatin to access the broken ends and various acetylases and deacetylases orchestrate this process [3]. One major acetyltransferase that facilitates repair via HR is TIP60/KAT5 which modifies nucleosome to facilitate localized chromatin remodeling [12–17]. KAT5 acetylates several residues of histone H4 including H4K16 which promote an open chromatin structure and facilitates long range resection of broken ends [12,14,18]. Impeding KAT5 activity inhibits both resection and HR mediated repair indicating that KAT5 may be involved in tipping the repair choice towards HR [19,20]. KAT5 mutations have been identified in human cancers highlighting its importance to accurate break repair [21–23].

PRMT5 is an arginine methyltransferase with pleiotropic functions in humans including in development and cancer [24,25]. PRMT5 mutations that promote cellular transformation have been identified in human cancers [26–28] and small molecule inhibitors targeting this enzyme have already been developed [29–32]. One of its functions is to methylate histones H2A, H4 and H3 in human cells. Another function targets the RUVBL1 subunit of the KAT5 complex which is methylated at R205. PRMT5 dependent RUVBL1 methylation is required for recruitment of the KAT5 complex to the breaks via interaction with histone H3K9me3 [33]. Thus, PRMT5 directly participates in orchestrating DSB repair.

In a previous report we characterized all PRMT5 mutations in human cancers and identified several amino acid substitutions in the active site of the enzyme [28]. Using *in silico* protein structure analysis, we also showed that certain high frequency PRMT5 mutations may destabilize the function of the enzyme. In another publication, we also characterized the PRMT5 and RUVBL1 and highlighted co-occurring mutations between these two genes [34]. Here, we extended these previous studies and analyzed genetic interactions between PRMT5 mutations and mutations in other genes involved in the DNA damage repair (DDR) pathway. The hypothesis is that characterization of these epistatic interactions will reveal how PRMT5 fits within the DDR genetic pathway.

## Materials and methods

### Mutation data accession

Mutation data for the genes studied here were downloaded from COSMIC (https://cancer.sanger.ac.uk/cosmic) [35]. Independent files with mutations reported in all cancers were downloaded for each gene in.csv format.

### Adjustment of mutation frequency by protein length

To control for gene length and remove the possibility that larger proteins will register more mutations, we divided the total mutations for each gene by its length. Multiple mutations at the same position were included so that mutation burden or hotspots within each gene can be revealed. For example, if a short gene shows a high mutation/gene length fraction, we interpret it to be highly mutated in human cancers. This could be due to many mutations occurring at different positions throughout the protein or hotspots (mutations occurring at same position).

### Calculation of mutation fraction in 41 cancers

The percent mutations (fraction) for every gene queried here was calculated by cancer type. For example, for adrenal gland, 28% of samples show ATM mutations, 0% ATR, 16% BRCA1, etc.

### cBioPortal exclusivity analysis

Co-occurrence or exclusivity between PRMT5 and DSB repair genes was calculated using the cBioPortal calculator (https://www.cbioportal.org) [36,37]. For this analysis, we used the “Curated set of non-redundant studies” with the genome profiles set for mutations, structural variants, and all copy number profiles.

### Mutation driver potential

Determination of mutation driver potential was done using the Cancer Related Analysis of Variants Toolkit (CRAVAT) (https://opencravat.org) [38], using the CHASMplus tool [39]. This tool calculates the probability of any point mutation having driver potential by generating a p-value.

### Gene expression

Gene expression was downloaded from COSMIC as Z-values. Z-values are normalized to control tissue and are interpreted as normal expression (Z between −2 and +2), over-expression (Z higher than +2), and under-expression (Z lower than −2) [40]. We note that gene expression profiles are only available for TCGA samples.

### Calculation of selection pressure

Selection pressure was calculated using the methods described in [41]. N, S, L_N_ and L_S_ values were first extracted then the C_N_/C_S_ score was computed (S9 Table). Chi-square values were calculated as deviations from 1 ((C_N_/C_S_-1)^2^/1). A p-value was then computed using an online calculator (https://www.socscistatistics.com/pvalues/chidistribution.aspx) with 1 degree of freedom and 0.05 significance level.

### Structural analysis methods

Structures were obtained from the Protein Data Bank or AlphaFold (see S6 Table for details). Mutations were made using the mutagenesis function in The PyMOL Molecular Graphics System software. Residues within 4 Å of the mutated residue were shown and polar contacts excluding solvent were shown. The electrostatic surface potentials were calculated and visualized using the APBS plug-in for PyMOL [42]. The CUPSAT tool within the BRENDA Enzyme Database website was used to calculate ΔΔG values, torsional stability, and overall protein stability from the provided structure file [43]. In some cases, as indicated in S7 Table, the structure files were truncated due to size limitations with the CUPSAT tool.

All figures were made in Photoshop.

## Results and discussion

### Mutation frequency of PRMT5 and DDR genes in human cancers

**Fig 1:**
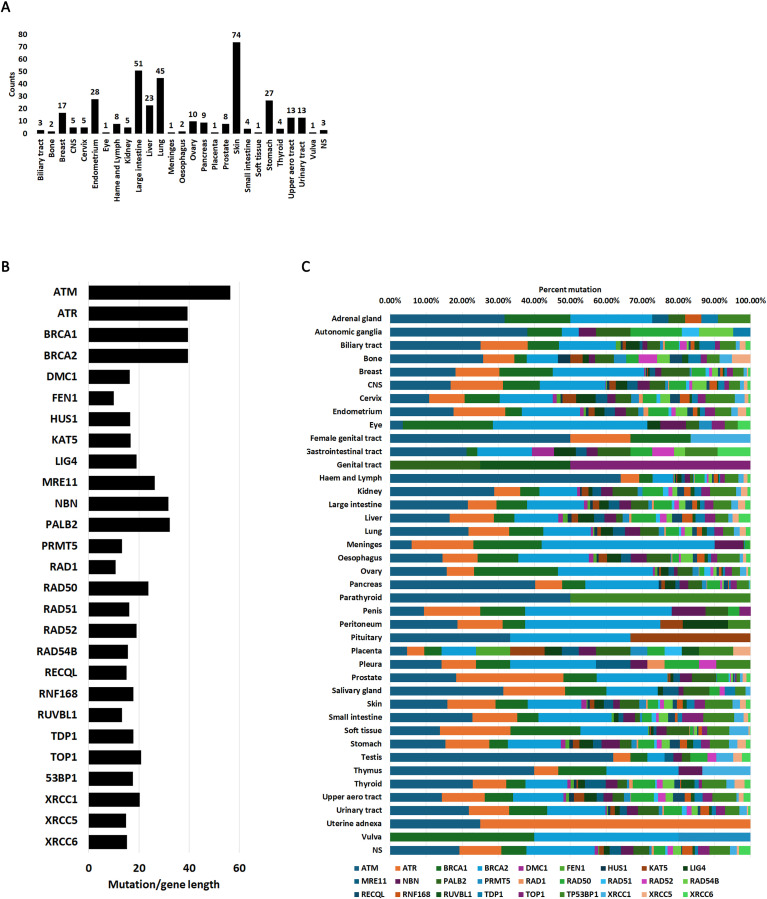
Mutation burden of 27 DNA damage repair genes in 41 cancer tissues. A. PRMT5 mutation counts (total tumors analyzed per cancer type) in all samples reported on COSMIC. B. Gene mutation frequency in all cancers adjusted by gene length C. Coding mutation occurrence in each gene in the 41 tissues queried. Complete data in S2 Table.

COSMIC reports PRMT5 mutations in nearly every cancer type (Fig 1A). Certain cancers such as large intestine, lung or skin have more mutations than other, but this is likely due to more samples reported. Nevertheless, PRMT5 mutation is endemic to human cancers highlighting the importance of this gene in cellular transformation and immortalization.

We chose 27 DNA damage repair genes for this analysis based on their roles in DSB repair and their interaction with PRMT5. We focused primarily on the KAT5 chromatin remodeler and associated proteins, the RAD52 epistatic group including BRCA1 and BRCA2 and several accessory helicases and nucleases. The checkpoint kinases ATM and ATR were also interrogated because PRMT5 has been shown to modulate ATM function through KAT5 regulation [33]. The function of these genes is summarized in S1 Table.

COSMIC reports both coding and non-coding mutations (S2 Table). Coding mutations occur in the translated regions while non-coding mutation can be intronic or in the 5’ and 3’ UTR. Although both types of mutations can destabilize gene function coding mutations are generally considered more deleterious than non-coding mutations because they directly affect protein structure and function. Coding mutations can occur in any residue and longer genes will generally accumulate more mutations than shorter genes. For example, analysis of cancer genomes has identified that certain long genes such as TTN (34350 residues) are highly mutated in breast cancer but their contribution to cancer development is still being explored [44]. In fact, these long genes can produce false positives [45]. The genes analyzed here were chosen based on their function in DSB repair and their roles in cancer are based on function rather than mutation frequency. Nevertheless, certain genes are much longer than others. For example, ATM has 3056 residues while RUVBL1 is 456 residues long. To control for gene length, we divided total coding mutations reported on COSMIC by gene length (Fig 1B).

When we interrogated all mutations in human cancers in the genes from S1 Table, we find that mutations have been reported in all genes and the highest mutation burden was in ATM, ATR, BRCA1 and BRCA2 despite being longer genes (Fig 1B). This was not unexpected because ATM is the major checkpoint signaling kinase of non-S-phase DSBs [46] while ATR facilitates repair of damage from replication stress [47]. BRCA2 is required for loading of the RAD51 recombinase to initiate homologous recombination repair [48]. BRCA2 is assisted by BRCA1 and other associated proteins [49]. RAD52 can also load RAD51 but its role is accessory to BRCA1-BRCA2 [50]. Mutations in BRCA1 and BRCA2 are endemic in human cancers, and they form negative epistatic interactions with RAD52 [51–53]. This observation highlights the importance of a functional machinery for RAD51 loading. Not unexpectedly, RAD51 mutations are rarer in human cancers. Mutations in the other DSB repair genes were also identified at various frequencies (Fig 1B). Mutations in all genes tested here were prevalent in all cancers reported (Fig 1C) with a few exceptions (e.g., uterine adnexa). However, this is due to small sample numbers for those cancers and should not be interpreted that those cancers are refractory to certain mutations. Taken together, these data show that DSB repair genes co-mutations are common in cancer cells.

### Mutation co-occurrence between PRMT5 and DSB repair genes

**Fig 2:**
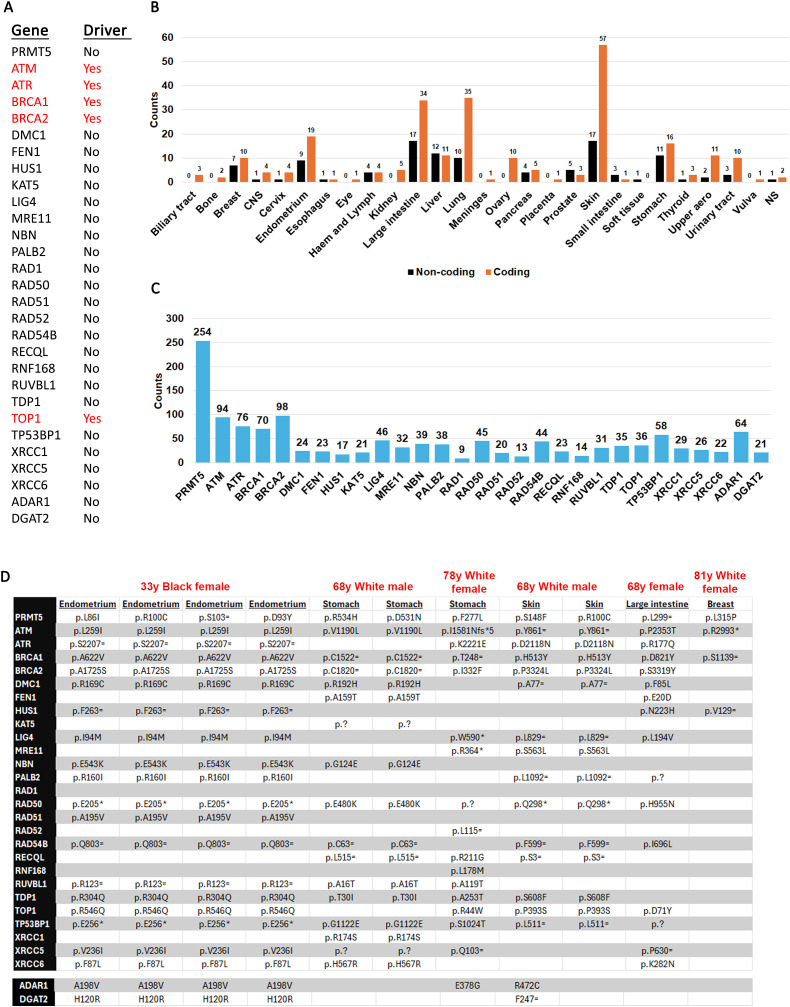
PRMT5 co-occurring mutations. **A**. Classification as driver or non-driver as reported on Integrative Oncogenomic. **B**. Counts of cancer tissues with co-occurring mutations between PRMT5 and at least one of the other genes. **C**. Counts of coding mutations in PRMT5 and other genes in samples where they co-occur. The PRMT5 bar represents co-occurring mutations (coding only) between PRMT5 and any of the other genes in the graph. The bars for the other genes represent co-occurring mutations (coding only) between that gene and PRMT5 (e.g., 91 ATM mutations co-occur with 91 other PRMT5 mutations). This graph includes both synonymous and non-synonymous point mutations. **D**. Cancer tissue samples where PRMT5 mutation co-occur with mutations in at least 50% of the other genes.

The prevalence of mutations in DSB repair factors suggests that mutations between PRMT5 and the genes studied here should co-occur. To investigate this, we used the cBioPortal mutual exclusivity calculator which generates statistical significance of co-occurrence (probability value). A probability value below 0.05 indicates co-occurrence. Indeed, this analysis showed that mutations between PRMT5 and all DSB repair genes studied here have a tendency to co-occur (S3 Table). Integrative Oncogenomics (https://www.intogen.org) [54] does not classify PRMT5 as a driver gene and only five of the other DSB repair genes are considered driver (Fig 2A). Classification of a gene as driver means that mutations are highly likely to promote cancer development while non-driver genes contribute less to cancer [54]. This observation is not unexpected because cells have redundant pathways for DSB repair and often mutations in more than one of these genes are required to cause improper repair [48,55].

To understand the nature of these co-occurring mutations we extracted amino acid coordinates from COSMIC (S4 Table). COSMIC lists mutations by sample number and we verified whether PRMT5 samples have mutations in the other DSB repair genes. Certain cancer types are characterized by more co-occurring mutations than others (Fig 2B). The endometrium, large intestine, lung and skin had the highest mutation burden between the genes tested. These cancers were also notable for having more coding mutations than non-coding (S5 Table) This suggests that endometrial, colorectal, lung and skin, cancers are more likely to have destabilized DSB repair machineries. Other cancers with higher percentage of co-occurring coding mutations were ovary, upper aerodigestive system and urinary tract. However, fewer co-occurring samples were reported in these cancers, so it is harder to draw meaningful conclusions.

We next investigated how many times mutations in other genes co-occur with PRMT5 (Fig 2C). For this analysis we looked only at coding mutations. As expected, coding mutations in BRCA1, BRCA2, ATM and ATR are most likely to co-occur with coding mutations in PRMT5. PALB2 co-occurring mutations were also high (38 counts). This was not unexpected because PALB2 functions in the BRCA1-BRCA2 axis to load RAD51 [56]. The MRN complex (MRE11, RAD50, NBN) is a break sensor that operates primarily during S-phase [57,58]. In a previous report we showed that human cancers are characterized by mutations in all three genes [59]. MRE11 and RAD50 evolved in bacteria and were retained in eukaryotes while NBN is found only in the eukaryotic domain [60] and is also the least conserved of the three genes [61]. Not unexpectedly because they work in the same complex, mutations in all three genes (MRE11, RAD50, NBN) co-occur with PRMT5 at relatively similar frequency. We also find that mutations in NHEJ genes (53 BP1, LIG4, XRCC5, XRCC6) or base excision repair (XRCC1), are also likely to occur. We also included two control genes that have not been reported to interact with PRMT5: ADAR1, an adenosine deaminase involved in mRNA editing; and DGAT2, a gene involved in fatty acid biosynthesis [62–65]. Mutations in these two control genes also co-occur with PRMT5 at generally similar frequency as any other gene that are not ATM, ATR, BRCA and BRCA2. Therefore, these data suggest that when analyzing pair-wise genetic interactions between PRMT5 and other DNA repair genes, it does not appear to form negative epistatic interactions.

Next, we catalogued those samples that had co-mutations in PRMT5 and at least 50% (13 genes or more) of the other genes studied here (Fig 2D, S4 Table). We identified one endometrial 33 year old Black female with four mutations (R534H, R100C, S103 = , D93Y), a 68 year old white male with two stomach mutations (R534H, D531N), a 78 year old white female with one stomach mutation (F277L), a 68 white male with two skin mutations (S148F, R100C), a 68 year old female (race unknown) with one large intestine mutation (L299=), and a 81 year old White female with one breast cancer mutation (L315P). The large intestine sample was notable for having only a silent PRMT5 mutation. In our previous report we analyzed PRMT5 mutation pathogenicity and driver potential using the CHASM artificial intelligence algorithm [28] and found that mutations at R100, D93Y, and R534H are likely pathogenic while the L315P is both pathogenic and likely driver. In silico protein structure analysis revealed that the L315P mutation affected binding of the SAM cofactor which acts as a methyl donor in the PRMT5 active site. Remarkably, the breast cancer sample is not characterized by high mutation frequency in the other genes which may suggest that significantly destabilizing the PRMT5 function may have a negative epistatic interaction with the other DSB repair genes (e.g., co-mutations may kill the cells). We note that mutations in the two control genes also co-occur with PRMT5 in these tissues at the same general frequency as most of the other DNA damage genes (e.g., PALB2, LIG4, NBN, etc.) (Fig 2D, S4D Table). This is expected because there should not be any negative genetic interaction between PRMT5 and ADAR1 or PALB2. Additionally, when we performed a less restrictive analysis with mutation between PRMT5 and 11 DNA damage repair genes, we uncovered two more samples (S4D Table). Both these samples had ADAR1 mutations and one had a DGAT2 mutation. Thus, the absence of mutations between PRMT5 and other genes such as KAT5 or HUS1 suggests a negative genetic interaction particularly when multiple DNA repair genes are destabilized in samples with PRMT5 mutations (see next sections). This could also be interpreted that the functions of KAT5 and 9-1-1 become essential when the DNA damage repair genes mutation burden increases.

To understand how other mutations in Fig 2D that have not been previously analyzed affect protein structure, we generated 3-D ribbon and electrostatic models of the mutant proteins (S1 Fig, S6 Table). Additionally, we extracted other physical properties using CUPSAT (S7 Table). Modeling of these mutations computationally identified a select few mutations that would be predicted to disrupt polar tertiary structure interactions; DMC1 R169C, PRMT5 S87Y, and PRMT5 S632L. CUPSAT analysis identified several mutations as significantly destabilizing to the protein structure (ΔΔG value of less than −1.0 kcal/mol); ATM L259I, PALB2 R160I, TDP1 R304Q, XRCC6 F87L, PRMT5 P515S, PRMT5 S87Y, and PRMT5 S632L. Only one mutation of those analyzed in CUPSAT, PRMT5 G466V, was found to significantly stabilize protein structure (ΔΔG value greater than +1.0 kcal/mol).

### Expression profiles of genes investigated here

**Fig 3:**
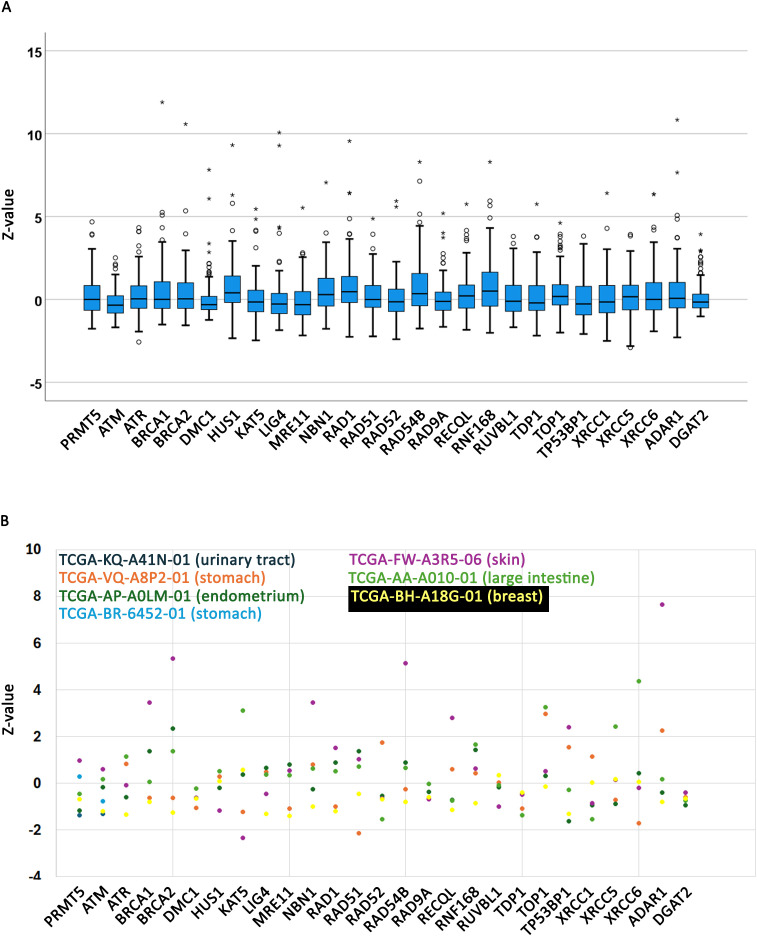
Expression profiles of genes studied here. **A**. Expression data (Z-values) for all TCGA samples discussed in this report. **B**. Expression profiles for the samples with high co-mutation burden shown in Fig 2D.

A caveat of the analysis performed in the previous section is that the genes and alleles reported here must be expressed to register a phenotype. COSMIC only reports expressions for certain TCGA samples which represent a subset of the data queried here (110/314, 35%). Gene expression data is reported on COSMIC as Z-scores, a value normalized to control. Z-scores are interpreted as normal expression for values between −2 and +2, under-expressed for values under −2, and over-expressed for values over +2 [40]. The expression Z-values for the genes analyzed here shows that their expression falls within the normal range (Fig 3A, S8A Table). Even though these data only represent 35% of the data they give a pan-cancer snapshot and there is no reason to believe that the other 70% of samples should behave differently. Thus, we conclude that the expression profiles of the allele studied here are normal.

Because most of the samples presented in Fig 2D and S4D Table are TCGA samples, we next extracted expression data for these samples with high burden of co-mutated alleles (Fig 3B, S8B Table). This analysis also reveals that expression profiles for these samples are within normal range. The only exception is the skin sample (TCGA-FW-A3R5-06) which shows elevated expression levels for some genes (BRCA1, BRCA2, NBN1, RAD54B, ADAR1). However, malignant melanoma are generally characterized by high chromosomal instability which is usually associated with increased chromosomal instability [66] which may require an increase in expression levels of DNA damage repair genes [67]. Other studies have also shown that an increase in DNA damage expression genes is an adaptation to radiation and other chemotherapeutic treatments [68–71].

### Selection pressure

Another confounding factor in the analysis presented here is selection pressure: the likelihood that a given mutation confers an advantage to the cancer cell. To identify any selection pressure acting on the genes studied here, we employed a technique ddescribed by Zhou et al which calculates selection based on the ration of nonsynonymous to synonymous substitution rate [41]. This analysis showed that most genes studied here are under neutral selection (S9 Table). Thus, it appears that co-occurring mutations between PRMT5 and these genes are not biased by selection pressure. The only outlier is ATM which appears to show positive selection (C_N_/C_S_ = 3.488; p value = 0.0128) and an argument could be made that.

### PRMT5 co-mutations with KAT5

**Fig 4:**
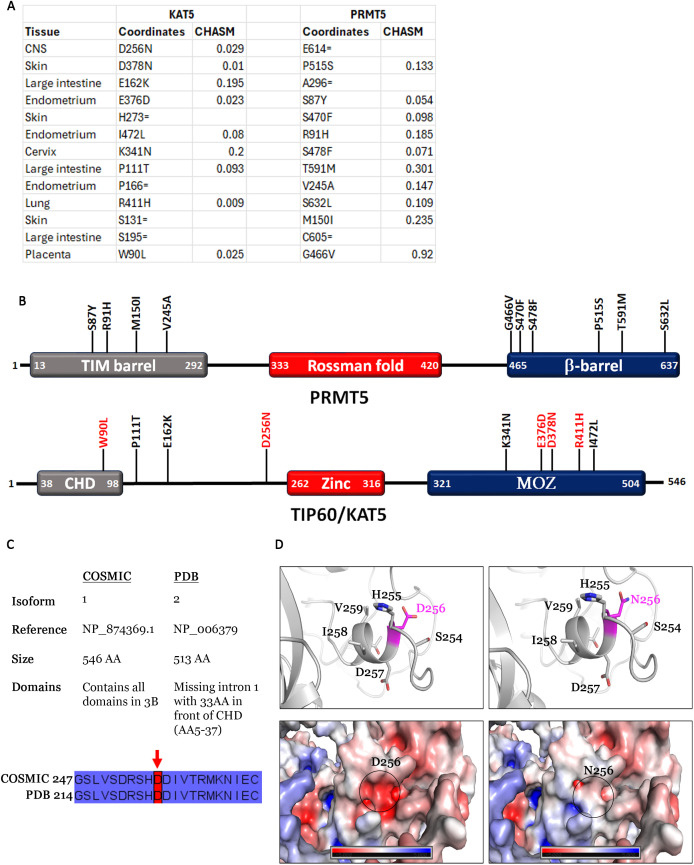
Co-occurring mutations between KAT5 and PRMT5. **A**. Tissues with KAT5 + PRMT5 co-occurring mutations and their corresponding CHASM p-values. **B**. PRMT5 and KAT5 diagrams showing positions of the co-occurring mutations. **C**. KAT5 isoforms as reported on COSMIC and PDB. COSMIC reports isoform 1 while PDB reports isoform 2. The difference between the two isoforms is that isoform 2 is missing 33amino acids form the N-terminus. All other domains align between the two isoforms and the position of the D256 residue is shown. **D**. Ribbon and electrostatic model of the D256 mutation.

We noticed that no KAT5 mutations co-occur with PRMT5 in the highly mutated samples shown in Fig 2D. KAT5 is a subunit of the TIP60 complex that has pleiotropic functions including DSB repair [72–74]. A previous report has shown that PRMT5 methylation of the RUVBL1 TIP60 subunit is required for recruitment of KAT5 to DSB breaks [33]. We note that there are several PRMT5 and RUVBL1 co-occurring mutations (Fig 2D) [34] but the absence of PRMT5-KAT5 co-occurring mutations suggests a negative epistatic interaction (e.g., destabilizing KAT5 in these samples that already have mutations in many DDR genes may be lethal). To understand the interaction between PRMT5 and KAT5, we investigated all samples with PRMT5 and KAT5 mutations regardless of whether or not any mutations occur in the other genes. This analysis found that of the total 254 PRMT5 mutation counts, only 15 co-occur with KAT5 (Fig 4A). Co-occurring mutations were not restricted to one cancer type. To understand the impact of these mutations on cellular transformation and immortalization, we calculated their driver potential using the CHASM artificial intelligence algorithm [38,39]. This algorithm classified several KAT5 mutations as driver (D256N, D378N, E376D, R411H, W90L) but none of the PRMT5 mutations had a significant p-value to be considered driver. The PRMT5 mutations cluster in the TIM barrel which binds MEP50 a protein that facilitates PRMT5 substrate interaction [75] and beta-barrel domains required for homodimerization [76] (Fig 4B). KAT5 mutations appear to occur throughout the protein sequence (Fig 4B). Since PRMT5 mutations are not driver and we have investigated them previously [28,34], we turned our attention to the effect of the KAT5 driver mutations on enzyme function.

In a previous publication we analyzed several KAT5 mutations and showed that the D378N and the E376D mutations may impact protein-protein interactions as they make contact with Epl1 in the *S. cerevisiae* NuA4 core complex, which corresponds to EPC1 in humans [21]. Here we identified a co-occurring mutation (D256N) that we did not previously map. COSMIC reports mutations using KAT5 isoform 1 (NP_874369.1) while PDB structures use isoform 2 (NP_006379) (Fig 4C). Isoform 1 is 546 AA while isoform 2 is 513 AA. The difference in size is due to alternative splicing of isoform 2 which does not include Exon1 and results in loss of 33 amino acids from the N-terminus (5-37) [77]. However, both isoforms include the CHD, Zinc and MOZ domains. We aligned the two isoforms and identified the corresponding D256 residue isoform 1 coordinates in isoform 2 (Fig 4C). Because all mutations were identified using the COSMIC isoform, for simplicity the structures in Fig 4D were labeled using isoform 1 coordinates. An analysis of the D256N mutation shows that it changes the local electrostatic surface potential of the protein and is predicted by CUPSAT to destabilize the protein structure by 2.42 kcal/mol (Fig 4D).

### PRMT5 co-occurring mutations with clamp component HUS1, RAD1, and RAD9

Our initial analysis showed that PRMT5 mutations are unlikely to co-occur with HUS1 and RAD1 (Fig 2C). HUS1 and RAD1 form a complex with RAD9 (so called 9-1-1 complex) which facilitates checkpoint activation through ATR and Chk1 during S-phase [78]. We found it interesting that ATR mutations do have a tendency to co-occur with PRMT5 but two members of the 9-1-1 complex appear to be interacting negatively with PRMT5 mutations. We therefore decided to investigate the nature of the mutations between the 9-1-1 complex and PRMT5 in more detail. Besides the 9-1-1 members (HUS1, RAD1, RAD9) which form a clamp analogous to PCNA [79] we also included RAD17, the 9-1-1 clamp loader [80]. Additionally, we investigated mutations in HUS1B and RAD9B. HUS1B is a paralog of HUS1 and can interact with RAD1 but not RAD9 [81]. Conversely, RAD9B, a paralog of RAD9 can interact with HUS1 and RAD1 to form the 9-1-1 complex but its expression appears to be restricted to testis and muscle cells but is highly expressed in tumor cells [82].

A review of all mutations reported on COSMIC revealed that mutations between PRMT5 and the 9-1-1 complex are indeed rare (Table 1, S8 Table). In fact, no sample had a co-mutation between PRMT5 and RAD9A or the RAD17 clamp loader. Co-mutations were also not found between PRMT5 and either of the RAD9B or HUS1B paralogs. We identified only 8 samples that had mutations between PRMT5 and either HUS1 or RAD1 but never both. This suggests that destabilizing both PRMT5 and the 9-1-1 complex may be refractory to cellular transformation and immortalization.

**Table 1:** Mutations between PRMT5 and checkpoint clamp members.

Cancer type	PRMT5	HUS1	RAD1	Other demographics^1^
Skin	S375F	M173I		73 year old male with malignant melanoma
Endometrium	R534H		R83*	47 year old female with endometrioid carcinoma
Liver	C42*	H231N		67 year old male
Endometrium	L637I		M1?^2^	69 year old female with endometrioid carcinoma
Large intestine	V547A		S198G	85 year old female with adenocarcinoma
Endometrium	L281I		T106I	84 year old female with endometrioid carcinoma
Skin	P528L	E66K		78 year old of unknown gender with basal cell carcinoma
Skin	W612L		Q6K	58 year old female with malignant melanoma

^1^As reported on COSMIC.

^2^This is a mutation in the first ATG codon (1A > G) which likely abrogates translation of the entire gene.

A crystal structure of the human RAD9-RAD1-HUS1 complex (PDB 3G65), was used to model mutations in HUS1 and RAD1 [83]. Residues were mapped onto the 3-D structure to determine if they were located in protein-protein interfaces. The HUS1 M173 residue is located on an alpha-helix that is in contact with RAD1 in the X-ray structure. RAD1 S198 is located in a protein-protein interface and is making key interactions with RAD9. Mutation of these two residues could destabilize these critical protein-protein interactions. In addition, the mutations were modeled to determine their effect on tertiary structure, electrostatic surface potential, and overall protein structure stability (S1 Fig and S7 Table). Only RAD1 S198G was found to disrupt tertiary polar interactions. Two mutations (HUS1 H231N and RAD1 T106I) were predicted by CUPSAT to be highly destabilizing to the protein structure.

## Conclusion

Genetic interactions between different DNA damage repair pathways have been exploited for therapeutic targets. For example, DNA double strand break repair by homologous recombination require the action of the RAD51 recombinase which can be loaded by either BRCA1-BRCA2-PALB2 or RAD52 [51,84,85]. Cells with BRCA2 mutations can be selectively killed by inactivation of RAD52 [86]. Considering that PRMT5 single molecule inhibitors have already been developed [87], understanding how the gene interacts with other repair pathways will elucidate potential therapeutic applications. The data analyzed here show that PRMT5 forms negative genetic interactions with KAT5 and components of the 9-1-1 complex when multiple DNA damage repair genes are destabilized. This suggests that in samples with high mutation burden, PRMT5, KAT5 and 9-1-1 could be therapeutically actionable (e.g., inhibiting the functions of these genes could kill the cells).

The data presented in this report should not be considered ripe for clinical applications. Rather, it is meant to inform potential therapeutic developments. We are also cognizant of the fact that these findings would need to be evaluated by direct analysis in living cells, but this is beyond the scope of this report. Nevertheless, these results should enhance our understanding of interactions among various genetic DNA damage repair mechanisms operating in human cells.

## Supporting information

S1 Fig Structural analysis of co-occuring mutations. PyMOL was used to generate ribbon structures of WT structure are shown on the left and the mutated structure on the right with the residue of interest shown in magenta sticks and residues within 4 Å shown in gray sticks. Polar tertiary structure interactions with the side chain of interest are shown in orange sticks and with gray dashed lines and labeled with their corresponding distances in Å. In cases where the available structure corresponds to a different isoform the corresponding amino acid numbering is indicated. Electrostatic surface potentials show negative charges in red (acidic), neutral in white, and positive charges in blue (basic). The location of the residue of interest is shown with a black circle.
(PDF)

S1 Table Function of DSB repair genes interrogated in this report. Functional information extracted from NCBI (https://www.ncbi.nlm.nih.gov/).
(PDF)

S2 Table Mutation frequency of PRMT5 and DSB repair genes. The file lists all mutations (coding and non-coding) as well as partitioned by mutation type (e.g., silent, point, InDel, etc.). The data have been computed to indicate gene mutation fraction in each cancer and each row adds up to 100%. Data downloaded from COSMIC.
(XLSX)

S3 Table Probability of mutation co-occurrence using the cBioPortal exclusivity calculator. (XLSX)

S4 Table Coordinates of mutations co-occurring between PRMT5 and DSB repair genes. **A**. A list of all PRMT5 samples and whether a mutation is present in the other genes tested. **B**. PRMT5 samples with mutations in at least five other DSB repair genes. **C**. PRMT5 samples with mutations in at least 50% of the other DSB repair genes (13 or more). **D**. Demographics of patients listed in C.
(XLSX)

S5 Table Sample counts with co-occurring mutations between PRMT5 and other genes. The table shows sample counts with any mutation, coding and non-coding (total), non-coding only and fraction of coding.
(XLSX)

S6 Table Citations for structures analyzed. For every mutation analyzed structurally in S1 Fig the table shows which structure was used, either with a PDB identifier or the AlphaFold identifier.
(PDF)

S7 Table Summary of structural analysis results of co-occurring mutations. Summary of PyMOL structural analysis results from S1 Fig and CUPSAT analysis. Truncated structures were used for CUPSAT analysis in some cases (indicated in table) due to size limits of the software.
(PDF)

S8 Table PRMT5 co-occurring mutations with 9-1-1 clamp components. The table shows all PRMT5 mutations but highlighted in yellow are those samples with PRMT5 coding mutations.
(XLSX)

S9 Table Selection coefficient and p-value for genes studied here. Selections coefficients were calculated as described in [41].
(XLSX)
