# Supplementary material for: PRMT5 genetic interactions with DNA double strand break repair genes
Source: PLoS One. 2025 Oct 9;20(10):e0331499. doi: 10.1371/journal.pone.0331499 (PMC12510555; doi:10.1371/journal.pone.0331499)
Supplement: S1 Fig — PyMOL was used to generate ribbon structures of WT structure are shown on the left and the mutated structure on the right with the residue of interest shown in magenta sticks and residues within 4 Å shown in gray sticks. Polar tertiary structure interactions with the side chain of interest are shown in orange sticks and with gray dashed lines and labeled with their corresponding distances in Å. In cases where the available structure corresponds to a different isoform the corresponding amino acid numbering is indicated. Electrostatic surface potentials show negative charges in red (acidic), neutral in white, and positive charges in blue (basic). The location of the residue of interest is shown with a black circle. (PDF) [file pone.0331499.s001.pdf]

Supplementary Figure 1. Structural analysis of co-occurring mutations.

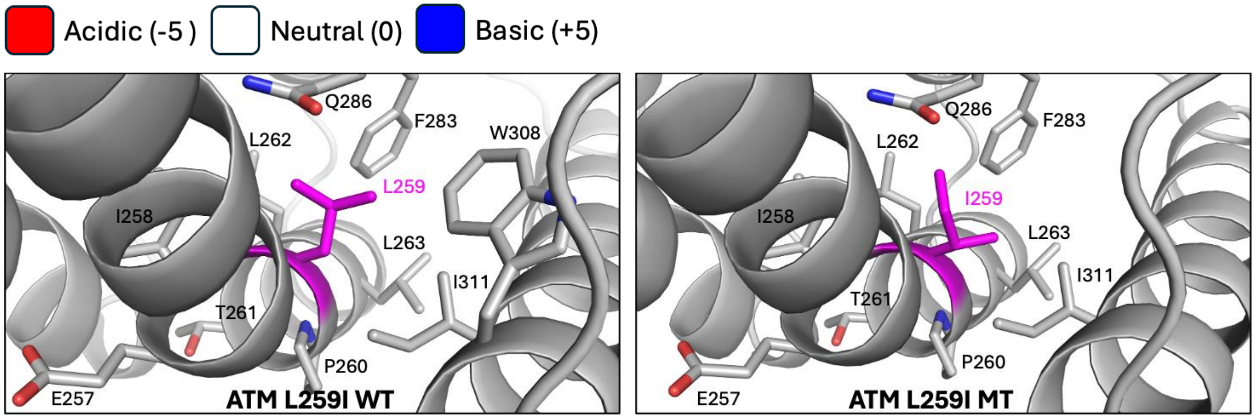

A: Ribbon model showing the ATM wild-type residue L259 and mutant I259.

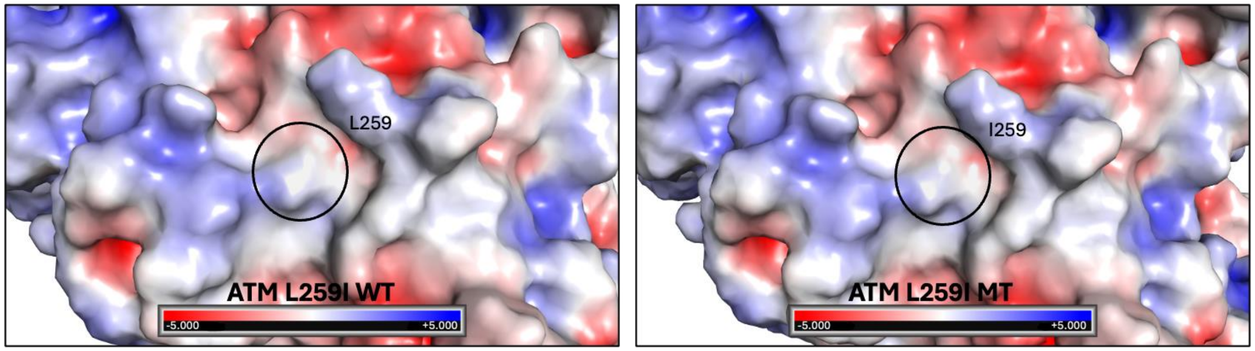

B: Electrostatic surface potentials of ATM wild-type residue L259 and mutant I259.

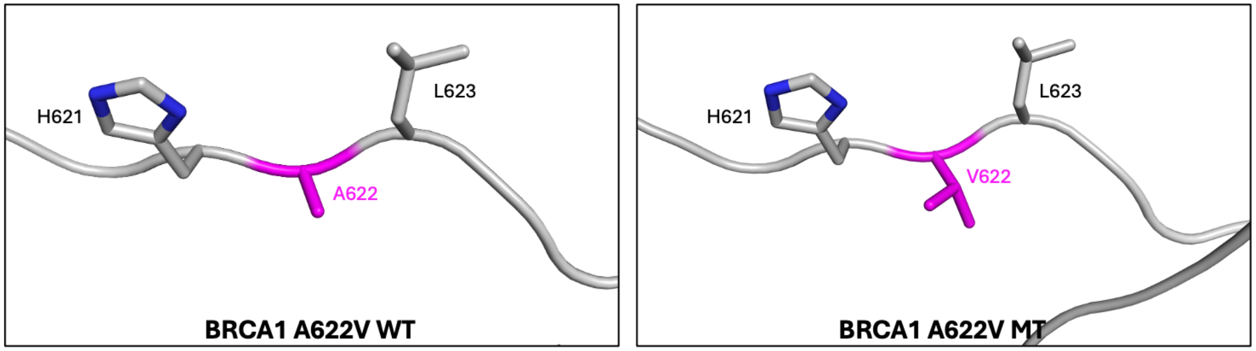

C: Ribbon model showing the BRCA1 wild-type residue A622 and mutant V622.

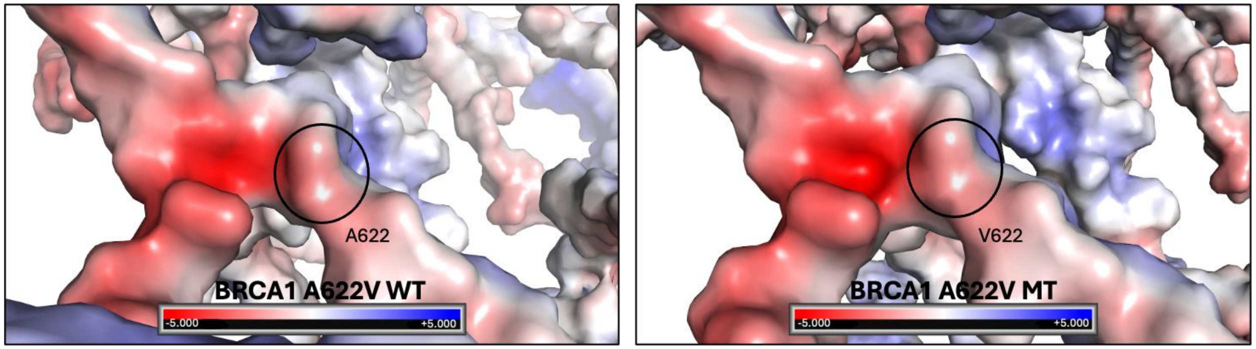

D: Electrostatic surface potentials of BRCA1 wild-type residue A622 and mutant V622.

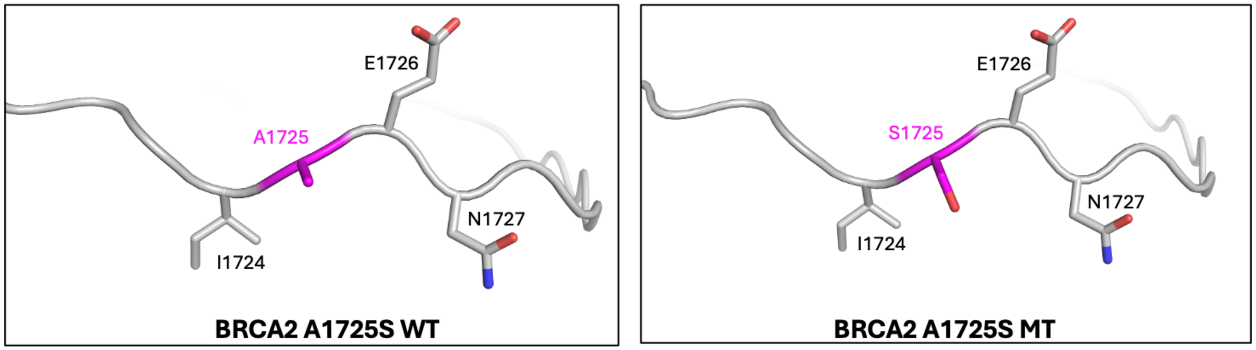

E: Ribbon model showing the BRCA2 wild-type residue A1725 (A121 in structure) and mutant S1725.

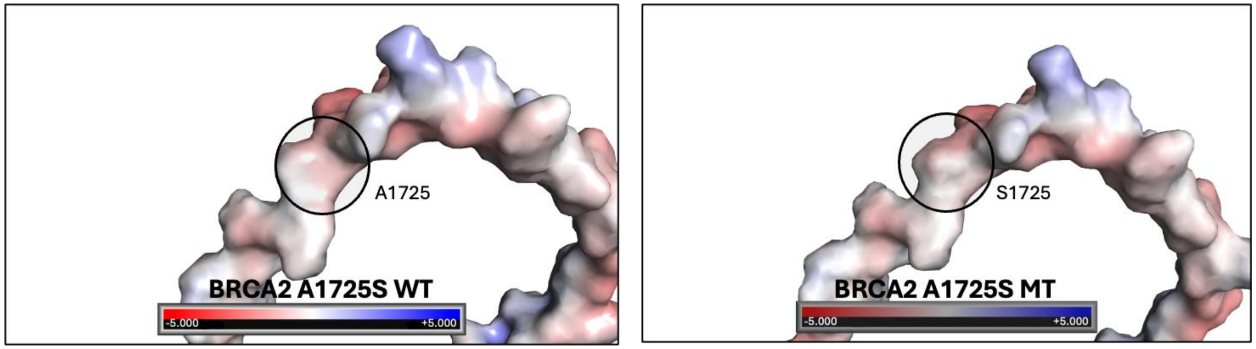

F: Electrostatic surface potentials of BRCA2 wild-type residue A1725 (A121 in structure) and mutant S1725.

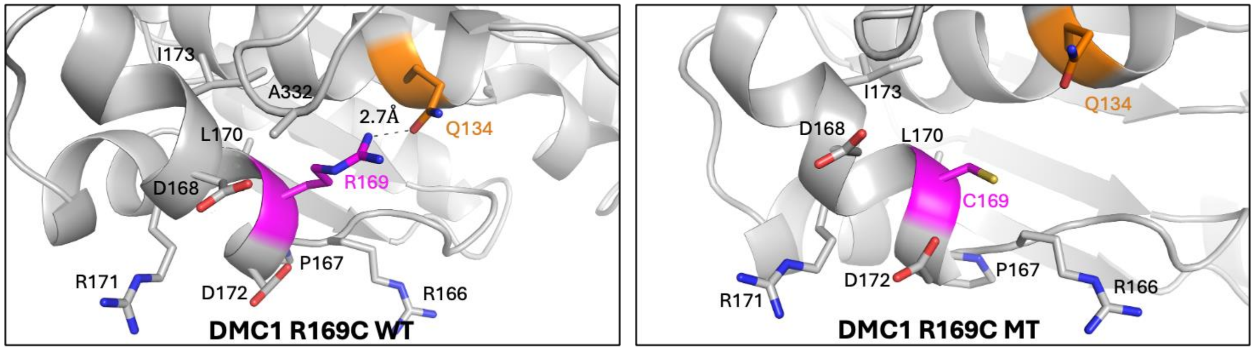

G: Ribbon model showing the DMC1 wild-type residue R169 and mutant C169.

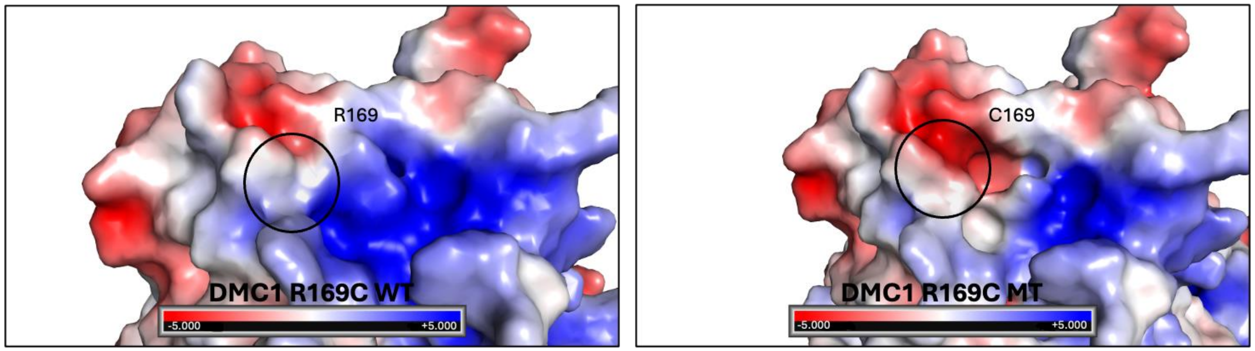

H: Electrostatic surface potentials of DMC1 wild-type residue R169 and mutant C169.

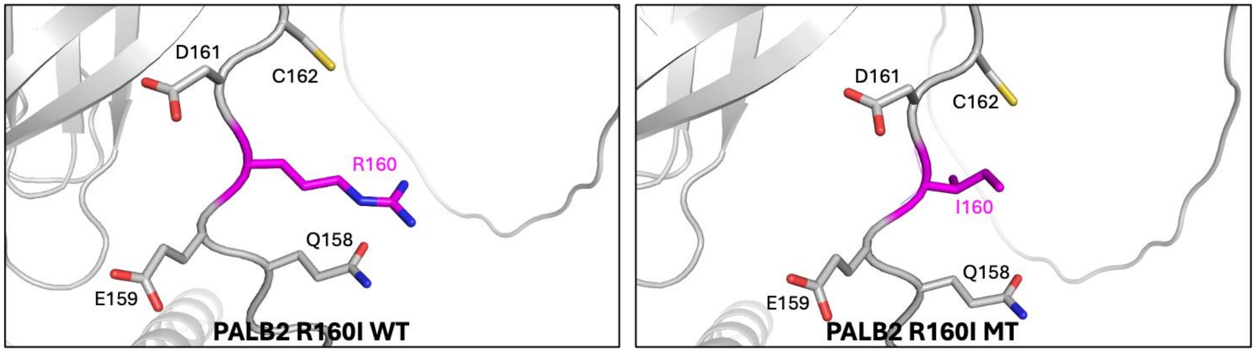

I: Ribbon model showing the PALB2 wild-type residue R160 and mutant I160.

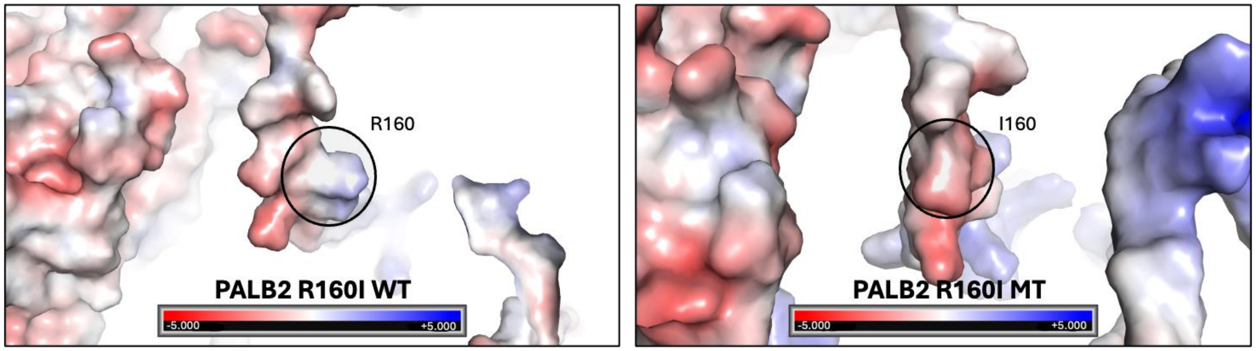

J: Electrostatic surface potentials of PALB2 wild-type residue R160 and mutant I160.

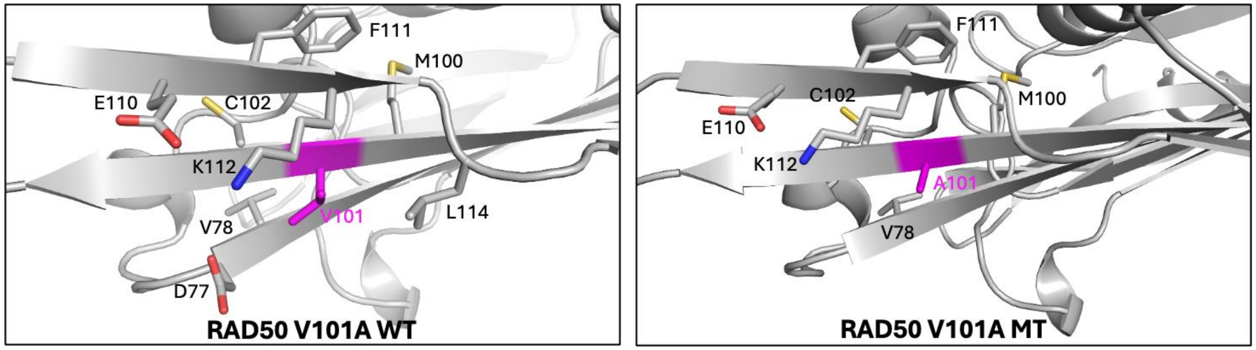

K: Ribbon model showing RAD50 wild-type residue V101 and mutant A101.

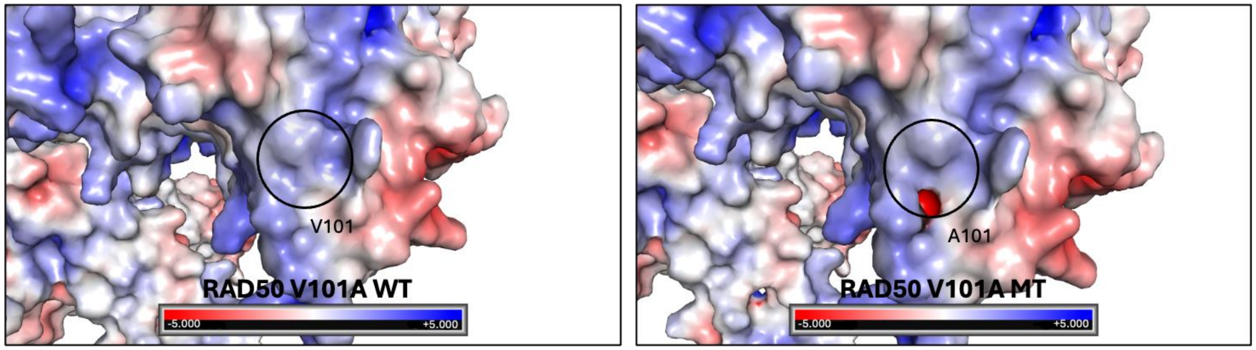

L: Electrostatic surface potentials of RAD50 wild-type residue V101 and mutant A101.

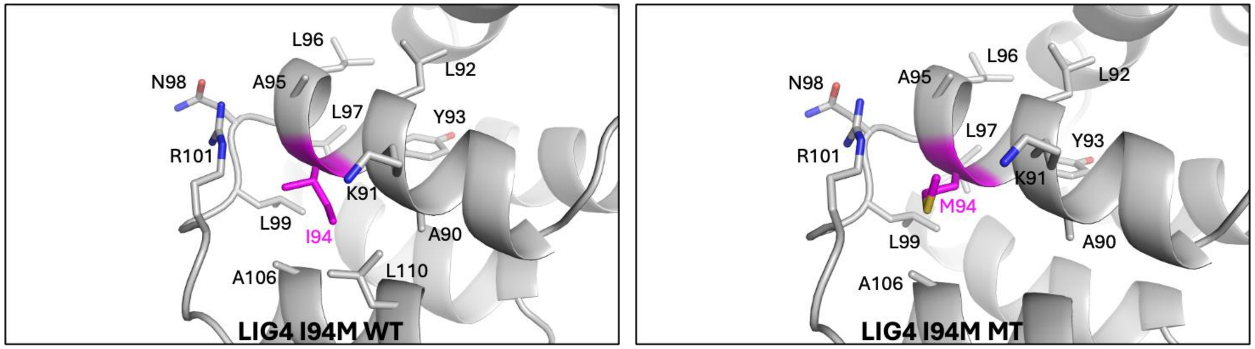

M: Ribbon model showing the LIG4 wild-type residue I94 and mutant M94.

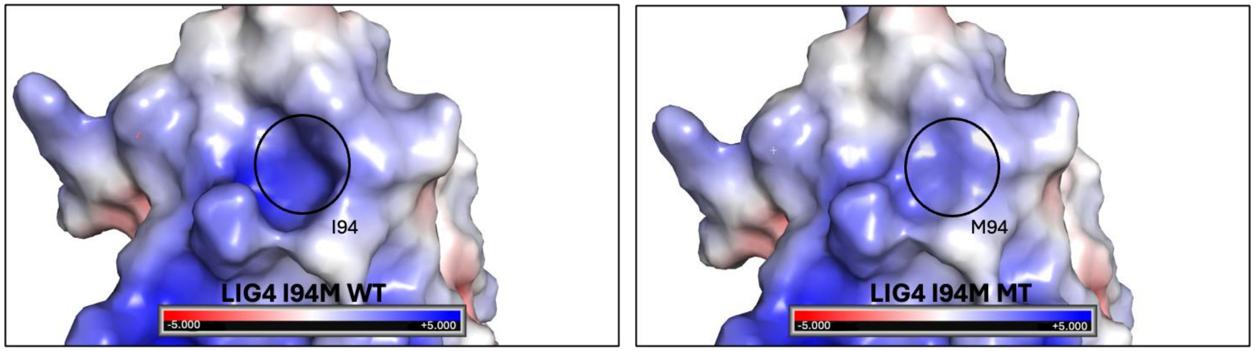

N: Electrostatic surface potentials of LIG4 wild-type residue I94 and mutant M94.

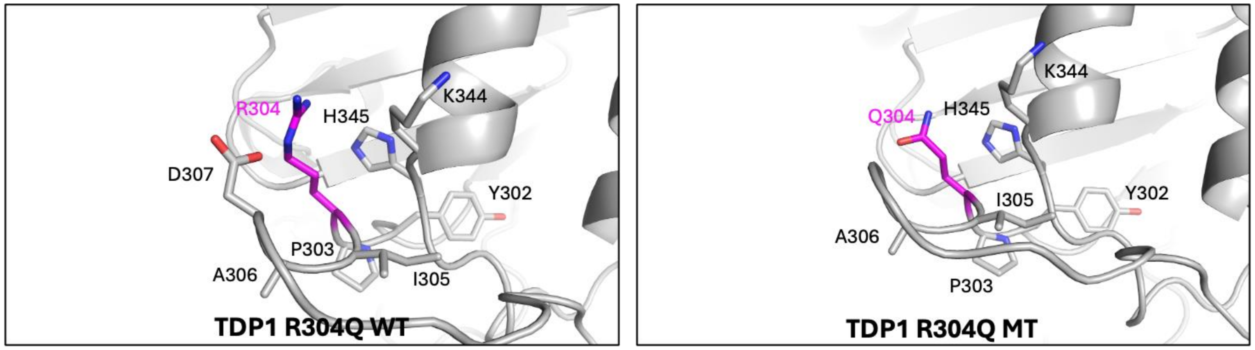

O: Ribbon model showing the TDP1 wild-type residue R304 and mutant Q304.

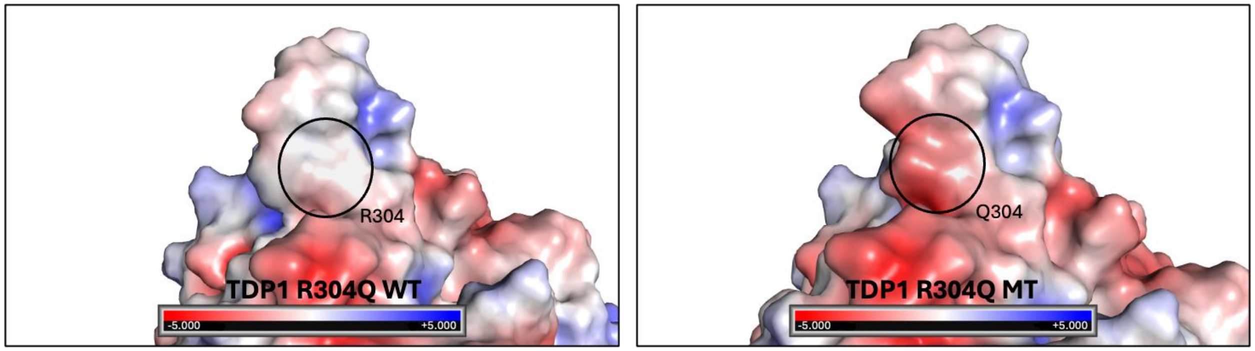

P: Electrostatic surface potentials of TDP1 wild-type residue R304 and mutant Q304.

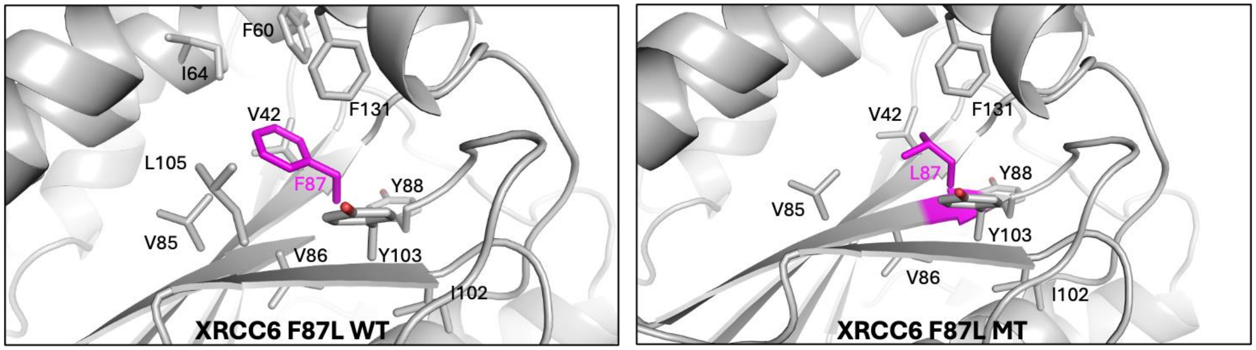

Q: Ribbon model showing the XRCC6 wild-type residue F87L and mutant L87.

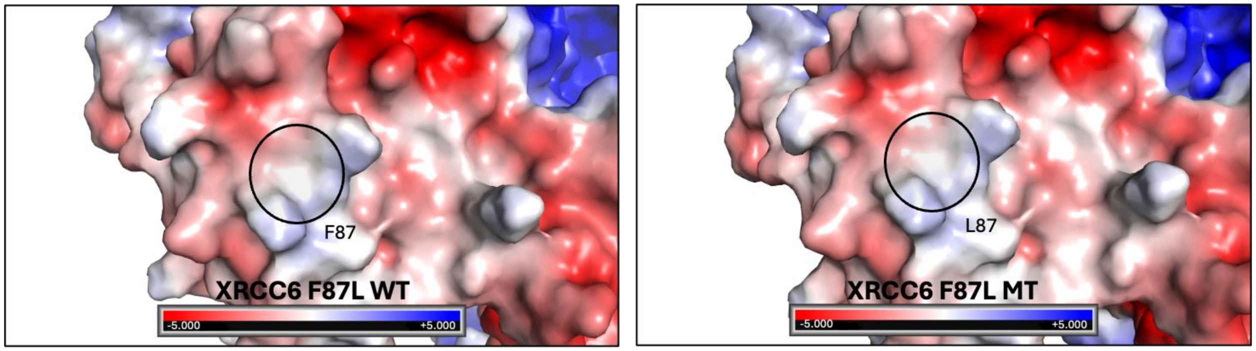

R: Electrostatic surface potentials of XRCC6 wild-type residue F87 and mutant L87.

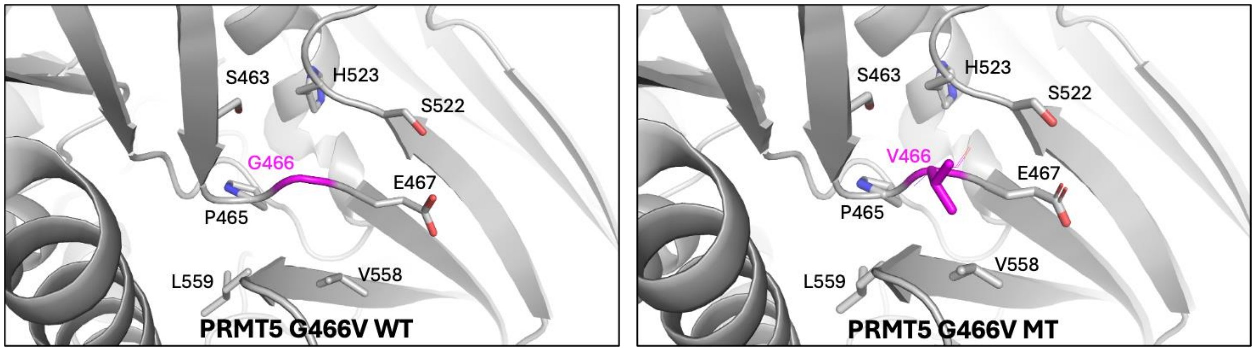

S: Ribbon model showing the PRMT5 wild-type residue G466 and mutant V466.

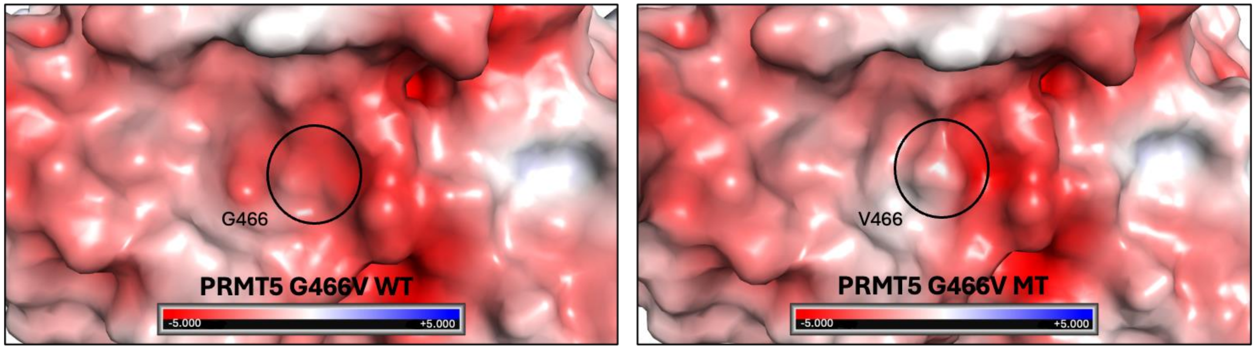

T: Electrostatic surface potentials of PRMT5 wild-type residue G466 and mutant V466.

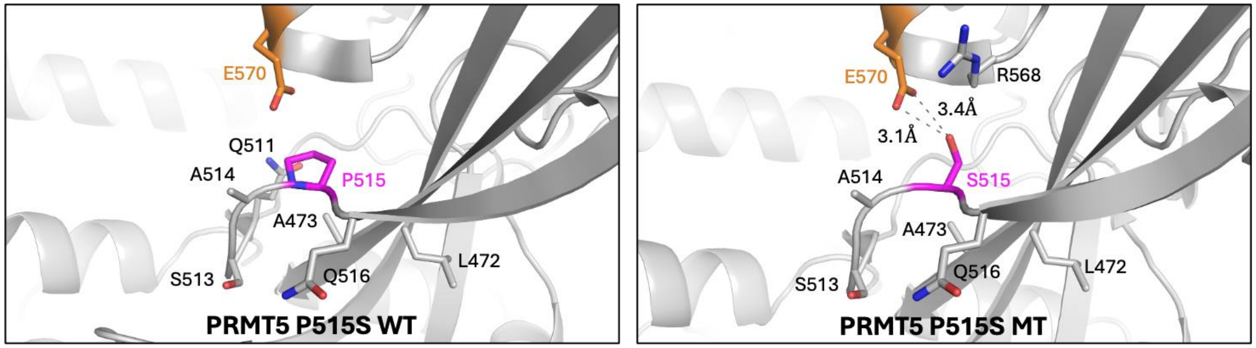

U: Ribbon model showing the PRMT5 wild-type residue P515 and mutant S515.

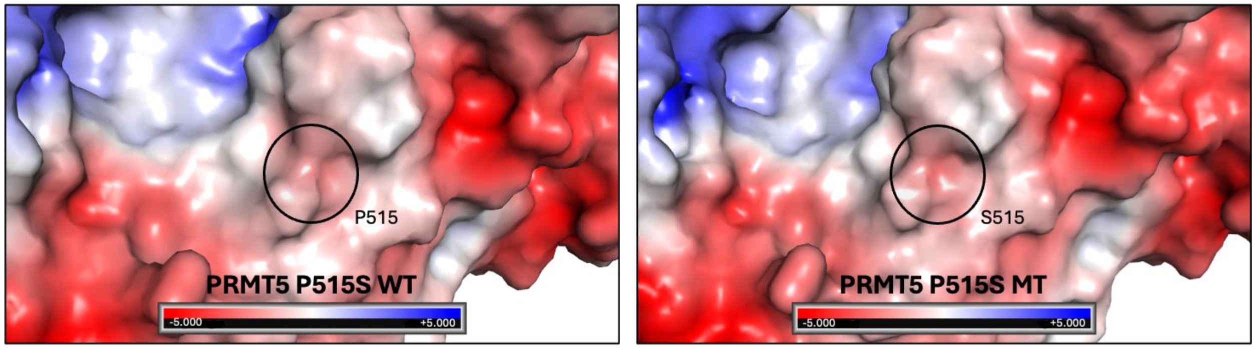

V: Electrostatic surface potentials of PRMT5 wild-type residue P515 and mutant S515.

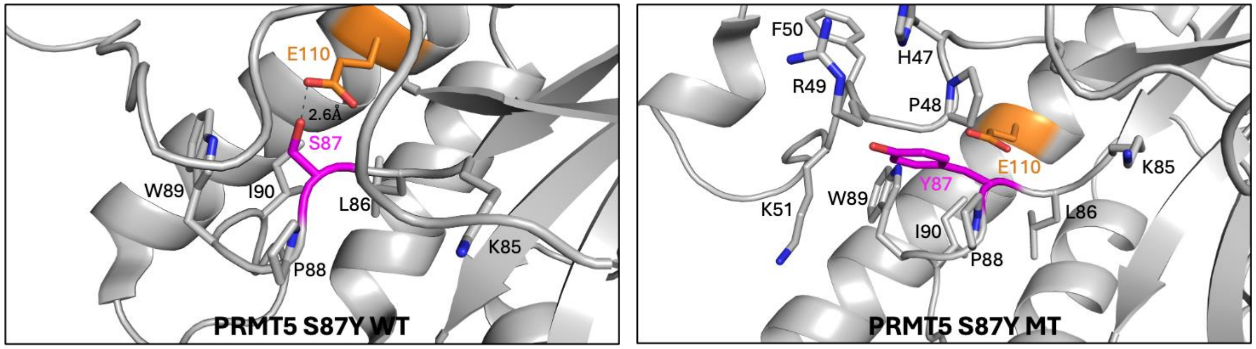

W: Ribbon model showing the PRMT5 wild-type residue S87 and mutant Y87.

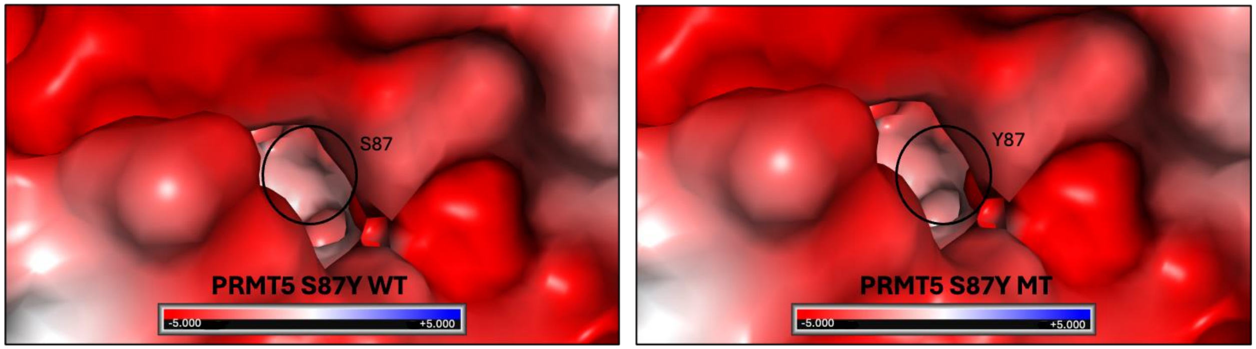

X: Electrostatic surface potentials of PRMT5 wild-type residue S87 and mutant Y87.

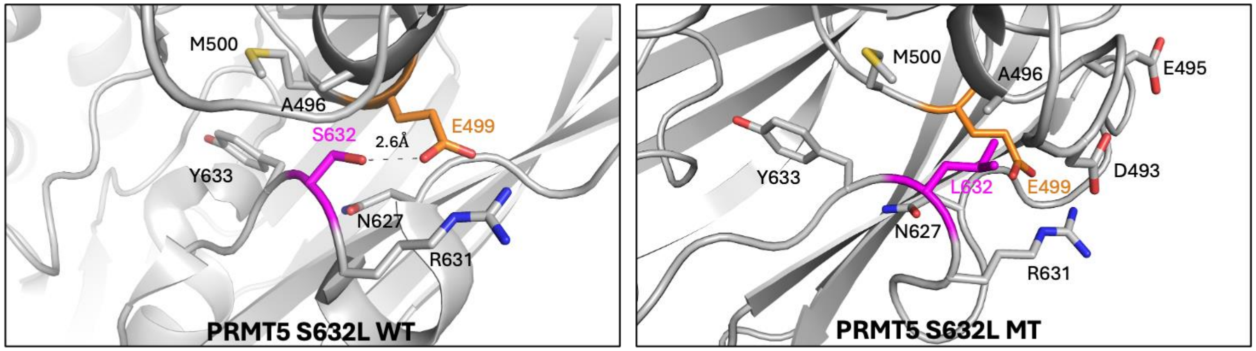

Y: Ribbon model showing the PRMT5 wild-type residue S632 and mutant L632.

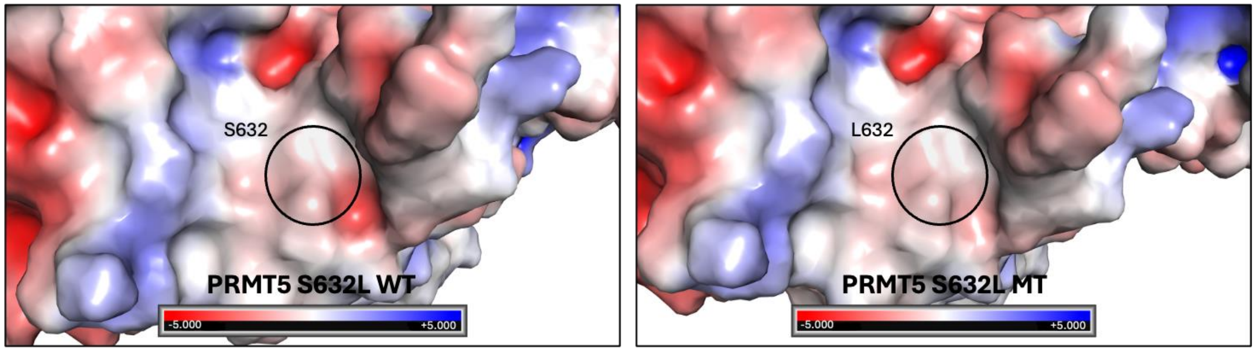

Z: Electrostatic surface potentials of PRMT5 wild-type residue S632 and mutant L632.

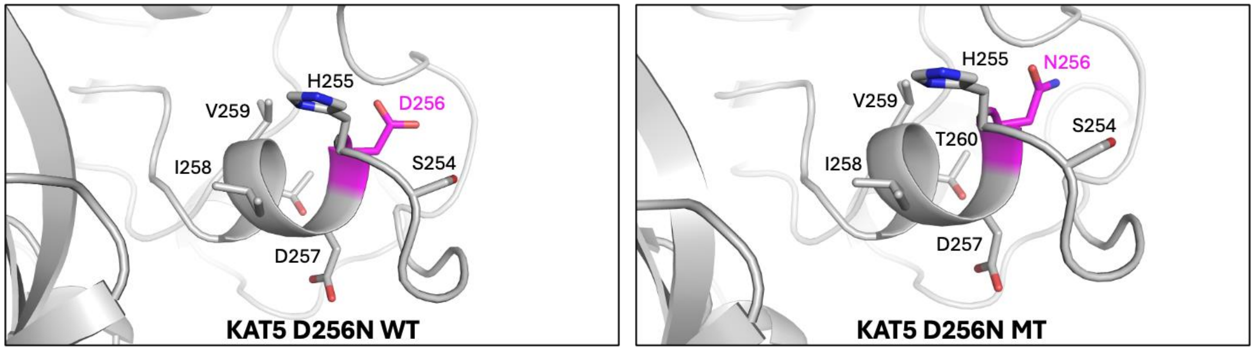

AA: Ribbon model showing the KAT5 wild-type residue D256 and mutant N256.

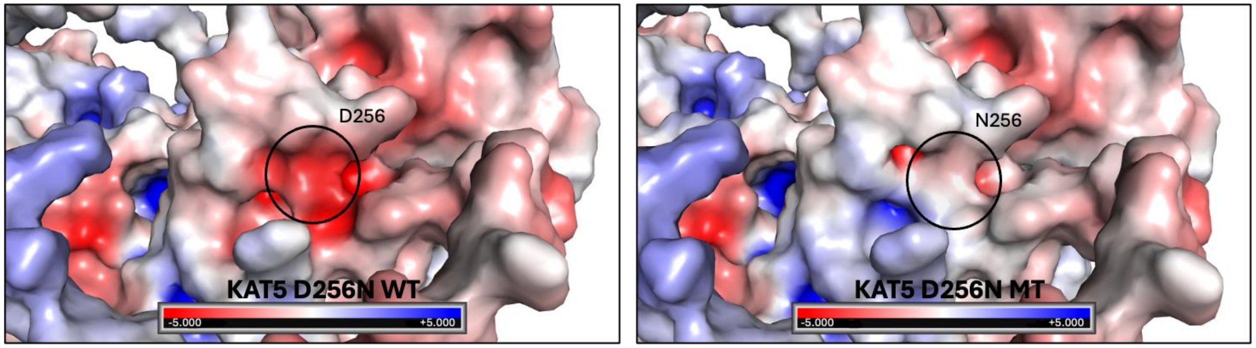

BB: Electrostatic surface potentials of KAT5 wild-type residue D256 and mutant N256.

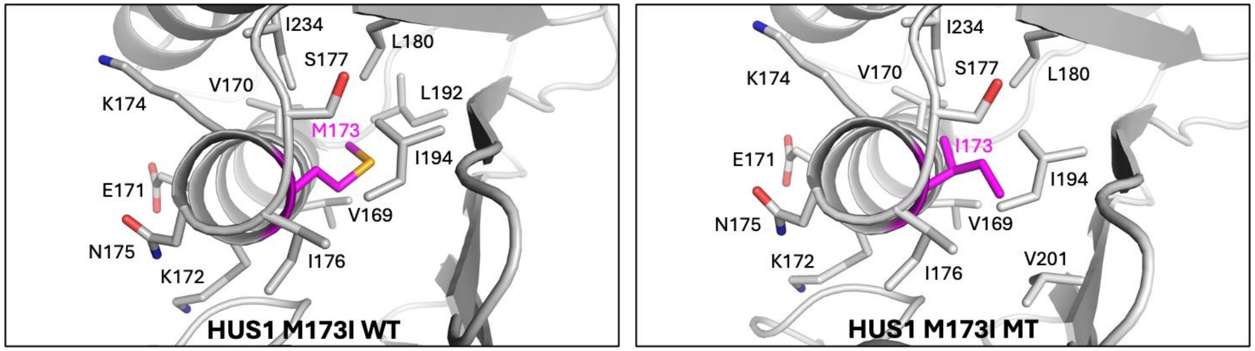

CC: Ribbon model showing the HUS1 wild-type residue M173 and mutant I173.

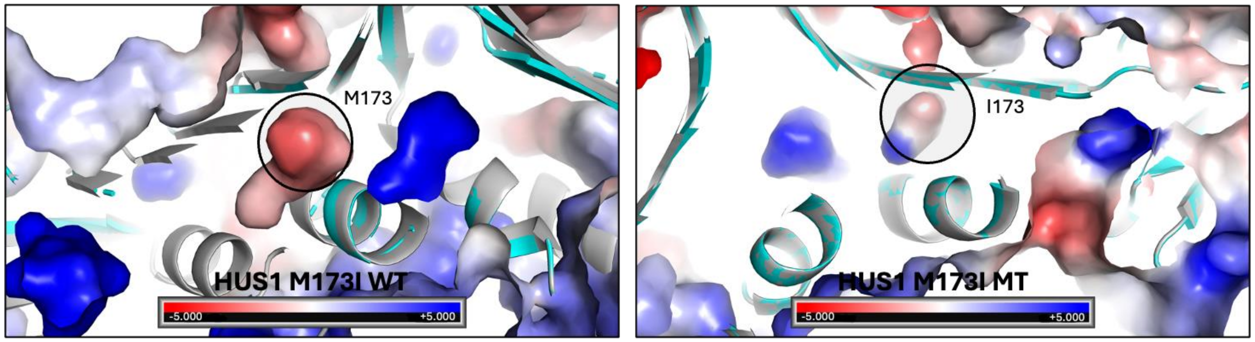

DD: Electrostatic surface potentials of HUS1 wild-type residue M173 and mutant I173.

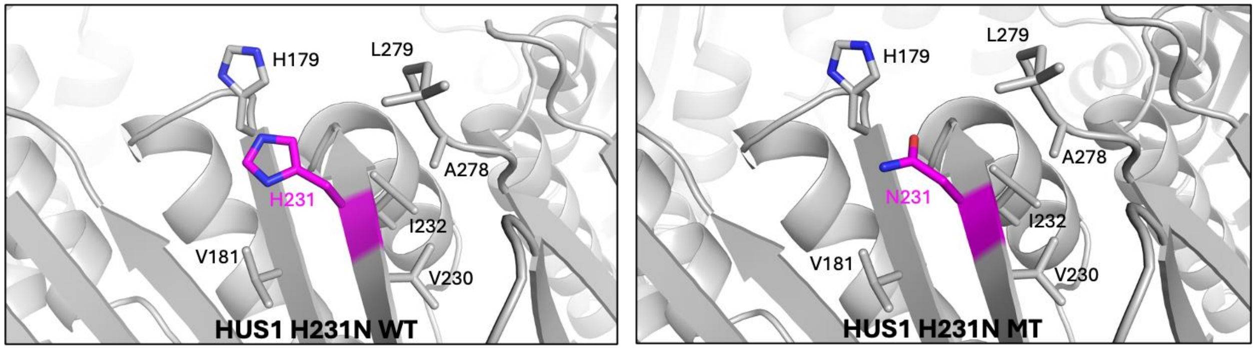

EE: Ribbon model showing the HUS1 wild-type residue H231 and mutant N231.

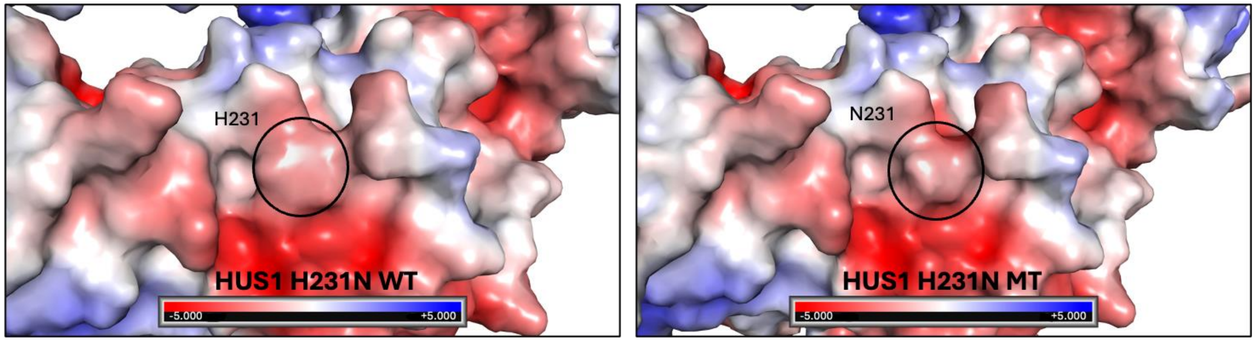

FF: Electrostatic surface potentials of HUS1 wild-type residue H231 and mutant N231.

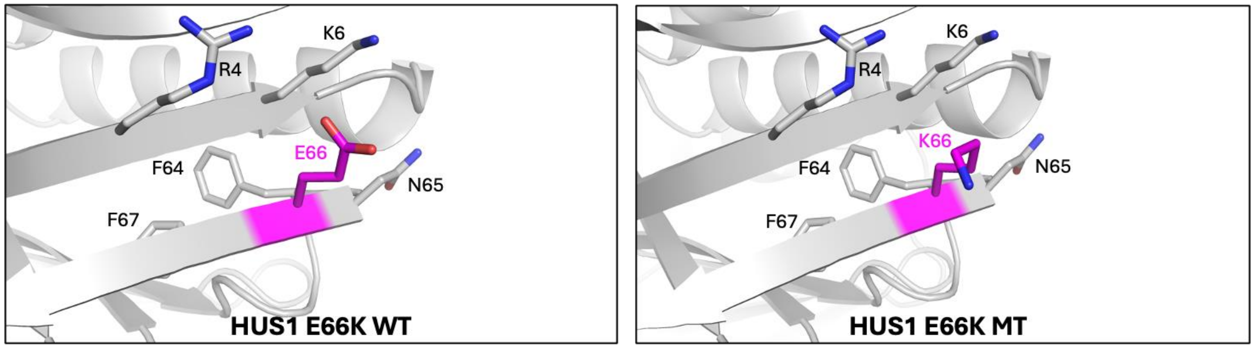

GG: Ribbon model showing the HUS1 wild-type residue E66 and mutant K66.

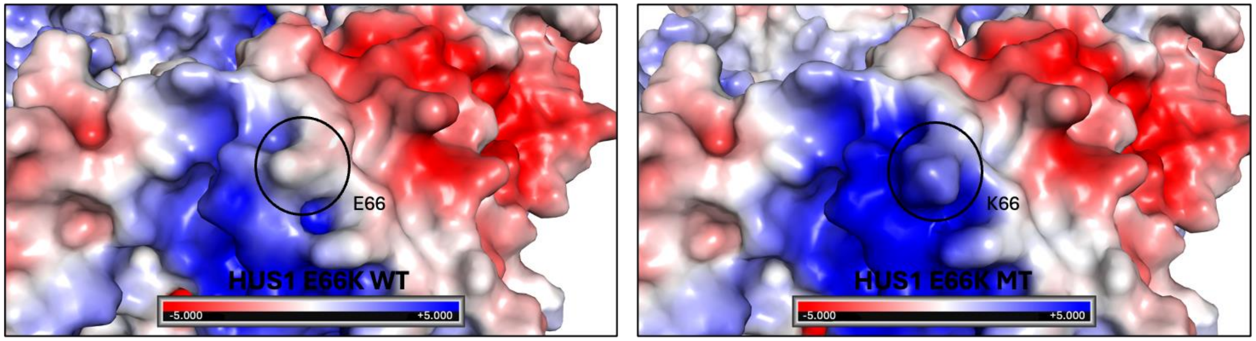

HH: Electrostatic surface potentials of HUS1 wild-type residue E66 and mutant K66.

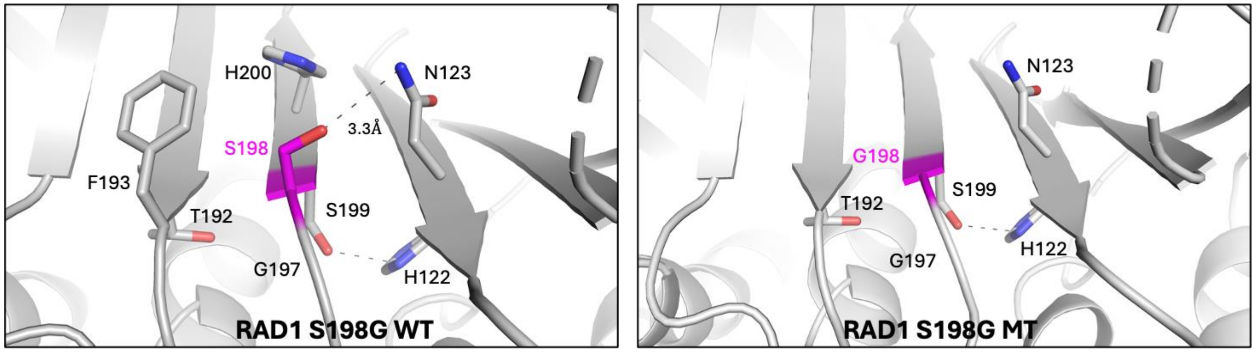

II: Ribbon model showing the RAD1 wild-type residue S198 and mutant G198.

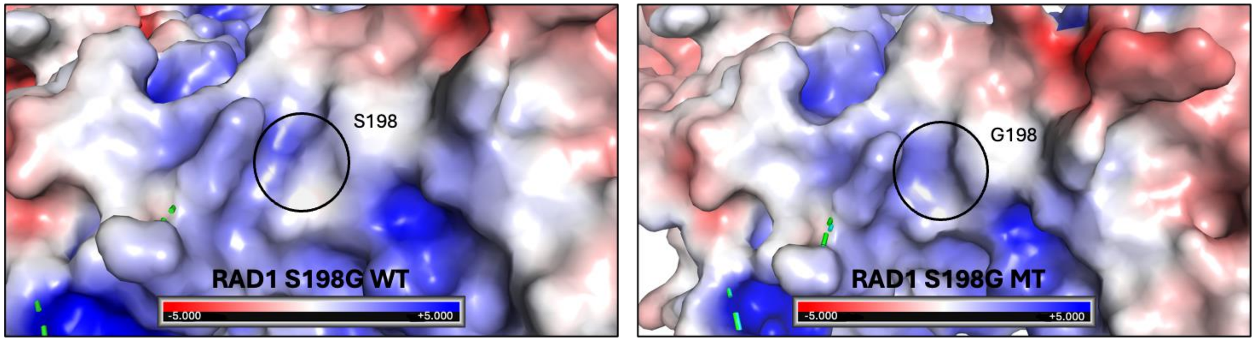

JJ: Electrostatic surface potentials of RAD1 wild-type residue S198 and mutant G198.

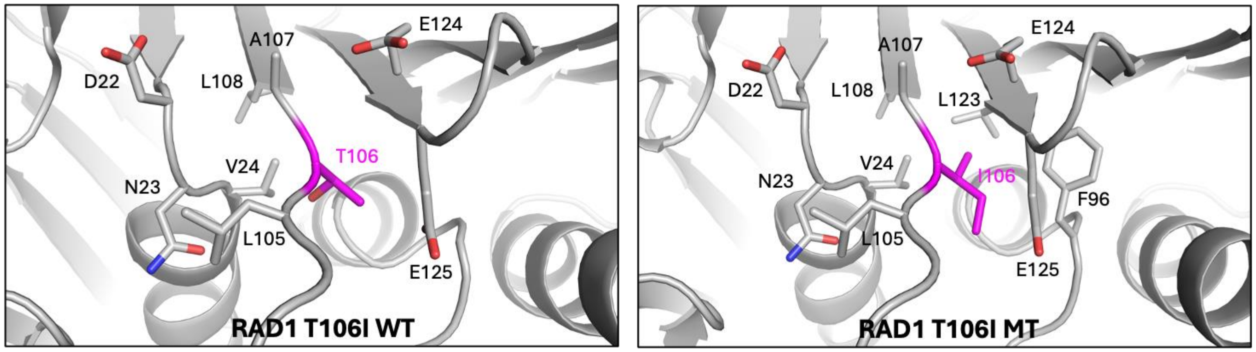

KK: Ribbon model showing the RAD1 wild-type residue T106 and mutant I106.

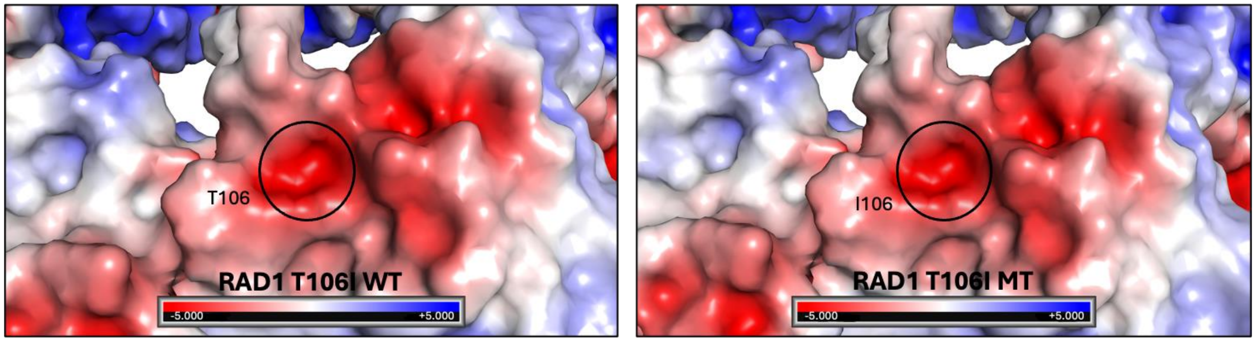

LL: Electrostatic surface potentials of RAD1 wild-type residue T106 and mutant I106.
