# Supplementary material for: PRMT5 genetic interactions with DNA double strand break repair genes
Source: PLoS One. 2025 Oct 9;20(10):e0331499. doi: 10.1371/journal.pone.0331499 (PMC12510555; doi:10.1371/journal.pone.0331499)
Supplement: S1 Table — Functional information extracted from NCBI (https://www.ncbi.nlm.nih.gov/). (PDF) [file pone.0331499.s002.pdf]

Supplementary Table 1. Function of genes interrogated in this report. Information from NCBI.

Gene	Function
ATM	Cell cycle checkpoint kinase activated in response to DSBs.
ATR	Cell cycle checkpoint kinase activated in response to DSBs arising during DNA replication.
BRCA1	Maintains genome stability. Tumor suppressor that interacts with histone deacetylase complexes, which plays a role in transcription, DSBs, and recombination.
BRCA2	Maintain genome stability in homologous recombination pathway for double-strand DNA repair and mediate binding to the RAD51 recombinase which functions in DNA repair.
DMC1	Repairs DSBs during mitosis and meiosis. Important for meiotic homologous recombination.
FEN1	Encodes proteins to remove 5' overhanging flaps in DNA repair and process 5' ends of Okazaki fragments in lagging strand DNA synthesis.
HUS1	Early checkpoint signaling involved in the cell cycle arrest in response to DNA damage. This protein forms a heterotrimeric complex with checkpoint proteins RAD9 and RAD1.
KAT5	A histone acetylase that has a role in DNA repair, apoptosis, and is thought to play an important role in signal transduction.
LIG4	V(D)J recombination and DSBs repair through nonhomologous end joining.
MRE11	Forms a complex with RAD50 required for nonhomologous joining of DNA ends.
NBN	Repairs DSBs and DNA damaged-induced checkpoint activation.
PALB2	A stable intranuclear localization and accumulation of BRCA2.
PRMT5	Catalyze the transfer of methyl groups to arginine, in target proteins that include histones, transcriptional elongation factors, and the tumor suppressor p53.
RAD1	Single stranded DNA binding activity and involved in DNA metabolic process.
RAD50	Repair DNA DSBs, cell cycle checkpoint activation, telomere maintenance, and meiotic recombination.
RAD51	Interacts with ssDNA-binding protein RPA and RAD52, homologous pairing and strand transfer of DNA, and cellular response to DNA damage.
RAD52	DNA double-strand break repair and homologous recombination.
RAD54B	Binds to double-stranded DNA and displays ATPase activity in the presence of DNA. Important for meiotic and mitotic recombination.
RECQL	DNA repair includes mismatch repair, nucleotide excision repair, and direct repair.
RNF168	Involved in protein-DNA and protein-protein interactions, and DNA DSB repair.
RUVBL1	Involved in multi-subunit transcriptional complexes and with protein complexes involved in both ATP-dependent remodeling and histone modification.
TDP1	Removes glycolate from single-stranded DNA containing 3-prime phosphoglycolate, repair of free-radical mediated DNA DSBs.
TOP1	Catalyzes the transient breaking and rejoining of a single strand of DNA, altering the topology of DNA.
TP53BP1	Promotes checkpoint signaling pathways for DNA damage like DSBs and NHEJ, limiting homologous recombination.
XRCC1	Repair DNA single strand breaks and base excision repair pathway.
XRCC5	Repair DNA DSBs by non-homologous end joining and the completion of V(D)J recombination events.
XRCC6	Repair of nonhomologous DNA ends required for DSB repair, transposition, and V(D)J recombination.
**Control genes**	
ADAR1	RNA editing through adenosine deamination. mRNA stability and function.
DGAT2	Synthesis of triglycerides. Involved in fatty acid biosynthesis
