# Supplementary material for: PRMT5 genetic interactions with DNA double strand break repair genes
Source: PLoS One. 2025 Oct 9;20(10):e0331499. doi: 10.1371/journal.pone.0331499 (PMC12510555; doi:10.1371/journal.pone.0331499)
Supplement: S6 Table — For every mutation analyzed structurally in S1 Fig the table shows which structure was used, either with a PDB identifier or the AlphaFold identifier. (PDF) [file pone.0331499.s007.pdf]

**Supplementary Table 6. Citations for structures analyzed.**

| Protein | PDB ID or AlphaFold ID | Citation PMID |
|---|---|---|
| ATM | 7SIC | 35076389 |
| BRCA1 | AF-P38398-F1-v4 | 34265844 |
| BRCA2 | AF-A0A248X3Z0-F1-v4 | 34265844 |
| DMC1 | 1V5W | 15125839 |
| LIG4 | 3W1G | 23523427 |
| PALB2 | AF-Q86YC2-F1-v4 | 34265844 |
| RAD50 | AF-Q92878-F1-v4 | 34265844 |
| TDP1 | 1JY1 | 11839309 |
| XRCC6 | 1JEY | 11493912 |
| PRMT5 | 4GQB | 23071334 |
| KAT5 | AF-Q92993-F1-v4 | 34265844 |
| HUS1 | 3G65 | 19446481 |
| RAD1 | 3G65 | 19446481 |
