# Supplementary material for: PRMT5 genetic interactions with DNA double strand break repair genes
Source: PLoS One. 2025 Oct 9;20(10):e0331499. doi: 10.1371/journal.pone.0331499 (PMC12510555; doi:10.1371/journal.pone.0331499)
Supplement: S7 Table — Summary of PyMOL structural analysis results from S1 Fig and CUPSAT analysis. Truncated structures were used for CUPSAT analysis in some cases (indicated in table) due to size limits of the software. (PDF) [file pone.0331499.s008.pdf]

Supplemental Table 7. Summary of structural analysis results of co-occurring mutations.

		PyMOL Analysis		CUPSAT Analysis			
**Protein**	**Mutation**	**Changes in Polar Interactions?**	**Changes in Electrostatic Surface Potential?**	**Predicted ΔΔG Value (kcal/mol)**	**Torsion Favorability**	**Overall Stability**	**AlphaFold structure? Truncated structure?**
ATM	L259I	No	None	-2.32	Favorable	Destabilizing	No, Not Truncated
BRCA1	A622V	No	None	-0.70	Favorable	Destabilizing	Yes, Truncated
BRCA2	A121S	No	None	0.94	Favorable	Stabilizing	Yes, Not Truncated
DMC1	R196C	Yes, Reduces Polar Interactions	Moderately basic to moderately acidic	0.60	Unfavorable	Stabilizing	No, Not Truncated
PALB2	R160I	No	Moderately basic to moderately acidic	-2.77	Unfavorable	Destabilizing	Yes, Not Truncated
RAD50	V101A	No	None	-0.97	Unfavorable	Destabilizing	Yes, Not Truncated
LIG4	I94M	No	Basic to moderately basic	-0.42	Favorable	Destabilizing	No, Not Truncated
TDP1	R304Q	No	Neutral to moderately acidic	-4.40	Unfavorable	Destabilizing	No, Not Truncated
XRCC6	F87L	No	None	-4.47	Unfavorable	Destabilizing	No, Not Truncated
PRMT5	G466V	No	Acidic to moderately acidic	1.17	Unfavorable	Stabilizing	No, Not Truncated
PRMT5	P515S	Yes, Increases Polar Interactions	None	-5.66	Favorable	Destabilizing	No, Not Truncated
PRMT5	S87Y	Yes, Decreases Polar Interactions	None	-4.32	Unfavorable	Destabilizing	No, Not Truncated
PRMT5	S632L	Yes, Reduces Polar Interactions	None	-1.89	Unfavorable	Destabilizing	No, Not Truncated

KAT5	D256N	No	Acidic to moderately acidic	-2.42	Unfavorable	Destabilizing	Yes, Not Truncated
HUS1	M173I	No	Acidic to moderately acidic	+0.05	Unfavorable	Stabilizing	No, Not Truncated
HUS1	H231N	No	None	-4.06	Favorable	Destabilizing	No, Not Truncated
HUS1	E166K	No	Neutral to basic	+1.56	Unfavorable	Stabilizing	No, Not Truncated
RAD1	S198G	Yes, Reduces Polar Interactions	None	-0.30	Unfavorable	Destabilizing	No, Not Truncated
RAD1	T106I	No	None	-4.21	Favorable	Destabilizing	No, Not Truncated
